# Verification of the Mode Decomposition Technique for Closely Distributed Modal Systems in the State Space Domain

**DOI:** 10.3390/s23167123

**Published:** 2023-08-11

**Authors:** Jungtae Noh, Jae-Seung Hwang

**Affiliations:** 1 Department of Architectural Engineering, Dankook University, Yongin 16890, Republic of Korea; jungtae.noh@dankook.ac.kr; 2 School of Architecture, Chonnam National University, Gwangju 61186, Republic of Korea

**Keywords:** state space model, closely spaced modes, mode decomposition, structure-tuned mass damper system, Operational Modal Analysis, high-rise building

## Abstract

This study aims to propose and validate the state space mode decomposition technique for precise mode separation of non-classical damping systems and closely distributed modal systems. To assess the reliability and applicability of this technique, a 40-story building with a tuned mass damper is investigated, and acceleration responses measured by the building’s health monitoring system are used for the verification of the technique. The mode separation results reveal that the separated modal power spectrum becomes distorted at neighboring natural frequency ranges when the performance index only considers the concentration of power spectral energy at the target natural frequency. However, by introducing an augmented performance index that includes a constraint condition to account for distortion, more accurate mode decomposition can be achieved.

## 1. Introduction

Structures with various shapes and diversified structural systems exhibit complicated dynamic behaviors. Wind load on high-rise structures leads to the idealized behavior characterized by mutually independent translational and torsional motions, and complex behavior where motions in different directions are combined. To understand the complex dynamic behavior, mode decomposition techniques that extract main modes are widely used for system identification [1,2,3,4]. The first-generation, operational modal identification method in the frequency domain is frequency domain decomposition (FDD). This technique introduced a novel approach aimed at effectively extracting mode shapes from measured responses and involves the utilization of singular value decomposition (SVD) applied to the cross-power spectral density (PSD) at an a priori estimated natural frequency of interest, employing peak picking methodologies for accurate identification [5]. Enhanced FDD (EFDD) represents a significant advancement in this field, as it introduces the application of inverse fast Fourier transform (IFFT) for PSD analysis near the target natural frequency. This innovative utilization can be used for estimating the damping ratio in the time domain, thereby enhancing the modal parameter analysis [6,7]. Furthermore, to address bias errors that may arise during damping estimation using EFDD, the frequency-spatial domain decomposition (FSDD) technique was introduced. Its successful application in civil engineering structures, including long-span bridges and stadium roofs, has proven its efficacy in manipulating damping estimation errors, leading to more reliable results [8]. Recent progress in the field includes the development of an advanced FDD method called frequency scale domain decomposition. This state-of-the-art approach applies SVD to the continuous wavelet transform of the PSD of the response to estimate modal parameters with greater precision and accuracy [9]. In addition, an alternate perspective on identifying modal properties has been explored that capitalizes on the orthogonality of filtered response vectors. The advantage of this method lies in its ability to dispense with the requirement of SVD after modal response decomposition, streamlining the analysis and simplifying the overall process [10].

The dynamic system’s nonlinear behavior can result in non-classical damping, observed not only in building structures but also in air and spacecraft structures under various loading conditions [11]. To tackle nonlinear identification, linear models such as the eigenvalue realization algorithm (ERA) have proven to be effective [12,13].

The Bayesian operational modal analysis (BAYOMA) method operating in the frequency domain and applicable for close modes has recently been developed based on an expectation-maximization algorithm that shows promise for simpler algorithm and computer coding [13]. Recent developments in the field of operational modal analysis (OMA) and blind source separation (BSS) have attracted considerable interest in separating modal response and estimating dynamic properties [6,14,15,16]. In recent years, there has been a surge in the popularity of BSS techniques for modal identification, effectively partitioning time series data into stationary and non-stationary components [17]. Among the high-order BSS methods, independent component analysis (ICA) has emerged as a prominent approach, facilitating decomposition of mixed signals into their respective linear transform matrix and source signals [18,19,20]. However, it is worth noting that the performance of ICA is somewhat constrained when dealing with modes characterized by low damping and closely positioned modal systems. To overcome this limitation, researchers have proposed an alternative solution known as second-order blind source separation (SOBSS), which has demonstrated superior effectiveness compared to high-order BSS algorithms [21,22,23]. This advancement holds great promise in addressing the challenges posed by complex and closely coupled modal behavior.

A major advantage of using OMA and BSS is that these approaches allow simple and effective estimation of the modal properties based on only the responses due to the ambient vibration other than a controlled artificial excitation to the structure as input. 

The mode decomposition methods, namely OMA and BSS, have been predominantly used in the MCK domain, which represents the equation of motions using the mass, damping, and method of the equation of motion as M, C, and K, respectively. However, these techniques have limitations in mode separation, especially when the modal damping is high, or modes are closely spaced. This results in incomplete mode decomposition, making it difficult to extract accurate modal information from the measured response [24]. To overcome these challenges, Hwang proposed a novel method called state space-based mode decomposition (SSBMD), which has been proven to be effective through numerical simulations [25,26]. Similar to conventional OMA techniques, SSBMD assumes white noise as an external force, which may not accurately represent the responses of an actual structure. To ensure reliable estimates of modal properties, it is recommended to conduct analytical simulations with enhanced frequency resolution. However, the performance of mode decomposition can still be affected by closely spaced neighboring modes, as the length of measurement response data can vary depending on the monitoring system used on-site.

This study aims to validate the optimized state space-based mode decomposition (OSSBMD) approach proposed by Hwang and Kim [26] through its application to the output signal from a benchmark building with closely spaced modes. A tuned mass damper (TMD) is a widely used vibration control device in tall buildings, which introduces non-classical damping that results in closely spaced modes. In this paper, the SSBMD framework with a new performance index is described, which can accurately separate modal responses in the state space domain. The closely spaced modes, in this study, are defined not only as neighboring natural frequencies but also those that cannot be separated using conventional techniques in the MCK domain due to the presence of non-classical damping. To validate the proposed method, the result of mode decomposition using monitoring data from a non-classically damped system is presented. The aim is to examine, experimentally, the effectiveness of the improved OSSBMD methodology in a high-rise building where a TMD is installed to control wind-induced vibration.

## 2. State Space Mode Decomposition

The equation of motion of the structure with *n* degree of freedom under the external load can be expressed as follows:

(1) $$M\ddot{x}+C\dot{x}+Kx=Ef\;$$

where *M*, *C,* and *K* represent the mass, damping, and stiffness of the system in size n×n, respectively, and *E* is the matrix indicating the location of the external load f. In this paper, the space in which all terms in Equation (1) are described is referred to as the MCK domain. However, a problem arises when the damping matrix is non-classical, that is the damping matrix cannot be expressed by a combination of the mass and stiffness matrix. In this case, mode separation may not be achieved in the MCK domain [27,28]. To address such a difficulty in mode decomposition, the equation of motion can be expressed in the state space domain as follows:

(2) $$\dot{z}=Az+Bf$$

where,

$$A=\left[\begin{array}{cc} 0 & I\\ -M^{-1}K & -M^{-1}C \end{array}\right]\;B=\left[\begin{array}{c} 0\\ M^{-1}E \end{array}\right]\;z=\left(\begin{array}{c} x\\ \dot{x} \end{array}\right).$$

Equation (2) indicates the transformed Equation (1) into the state space domain by the linear combination of the state variables z of displacement and velocity and the differential variable $\dot{z}$ of velocity and acceleration. The eigenvalue problem in the state space domain with respect to the system matrix *A* can be expressed as follows:

(3) AΨ=ΨΛ

where Λ and Ψ are the eigenvalue and the eigenmatrix, respectively as follows:

(4) $$\Lambda =\left[\begin{array}{ccccc} \lambda_{1} & & & & \\ & \lambda_{1}^{*} & & 0 & \\ & & \ddots & & \\ & 0 & & \lambda_{n} & \\ & & & & \lambda_{n}^{*} \end{array}\right]\cdots \Psi =\left[\Psi_{1},\Psi_{1}^{*},\cdots \Psi_{n},\Psi_{n}^{*}\right]$$

The eigenmatrix consists of the eigenvectors corresponding to the eigenvalues and is composed as shown in Equation (4). The superscript “*” indicates the complex conjugate. 

The state variable z can be transformed into a newly defined modal response in the state space domain as:

(5) $$z=\Psi q\;q=\Psi^{-1}z$$

where q is the response vector in the modal space. Substituting Equation (5) into Equation (2) gives:

(6) $$\dot{q}=\Lambda q+\Psi^{-1}Bf$$

The major problem with the mode decomposition using Equation (5) is that the resultant modal response has a complex number. This issue can be resolved by transforming the eigenproblem using the conjugate response as follows:

(7) $$\left[\begin{array}{cc} 0 & 1\\ -\omega_{i}^{2} & -2\xi_{i}\omega_{i} \end{array}\right]\;\left[\begin{array}{cc} 1 & 1\\ \lambda_{i} & \lambda_{i}^{*} \end{array}\right]=\left[\begin{array}{cc} 1 & 1\\ \lambda_{i} & \lambda_{i}^{*} \end{array}\right]\;\left[\begin{array}{cc} \lambda_{i} & 0\\ 0 & \lambda_{i}^{*} \end{array}\right]\;$$

where $\lambda_{i}=-\xi_{i}\omega_{i}+i\sqrt{1+\xi_{i}}\;\omega_{i}^{2}\;$. 

The general eigenproblem using Equation (7) is given as:

(8) $$\Lambda_{m}\;T=T\Lambda$$

$$\Lambda_{m}=\left[\begin{array}{cccccccc} 0 & 0 & 0 & 0 & 1 & 0 & 0 & 0\\ 0 & 0 & 0 & 0 & 0 & 1 & 0 & 0\\ 0 & 0 & 0 & 0 & 0 & 0 & \ddots & 0\\ 0 & 0 & 0 & 0 & 0 & 0 & 0 & 1\\ -\omega_{1}^{2} & 0 & 0 & 0 & -2\xi_{1}\omega_{1} & 0 & 0 & 0\\ 0 & -\omega_{2}^{2} & 0 & 0 & 0 & -2\xi_{2}\omega_{2} & 0 & 0\\ 0 & 0 & \ddots & 0 & 0 & 0 & \ddots & 0\\ 0 & 0 & 0 & -\omega_{n}^{2} & 0 & 0 & 0 & -2\xi_{n}\omega_{n} \end{array}\right]\;T=\left[\begin{array}{cccccccc} 1 & 1 & 0 & 0 & 0 & 0 & 0 & 0\\ 0 & 0 & 1 & 1 & 0 & 0 & 0 & 0\\ \vdots & \vdots & \vdots & \vdots & \ddots & \ddots & \vdots & \vdots \\ 0 & 0 & 0 & 0 & 0 & 0 & 1 & 1\\ \lambda_{1} & \lambda_{1}^{*} & 0 & 0 & 0 & 0 & 0 & 0\\ 0 & 0 & \lambda_{2} & \lambda_{1}^{*} & 0 & 0 & 0 & 0\\ \vdots & \vdots & \vdots & \vdots & \ddots & \ddots & \vdots & \vdots \\ 0 & 0 & 0 & 0 & 0 & 0 & \lambda_{n} & \lambda_{n}^{*} \end{array}\right]$$

The eigenvalue in Equation (8) is rewritten as:

(9) $$\Lambda =T^{-1}\Lambda_{m}T$$

Substituting Equation (9) into Equation (3) gives:

(10) $$\Lambda \;\Psi \;T^{-1}=\Psi \;T^{-1}\;\Lambda_{m}$$

In Equation (10), $\Psi \;T^{-1}$ is the transformation matrix which transforms the system matrix *A* to $\Lambda_{m}$ and consists of real number elements. Equation (10) is rewritten as:

(11) $$A\;T_{p}=T_{p}\;\Lambda_{m},\;T_{p}=\Psi \;T^{-1}\;$$

where $T_{p}$ is the unknown real-number transformation matrix to be used to transform into a system matrix expressed with the natural frequency $\omega_{i}$ and damping ratio $\xi_{i}$. As shown in Equation (5), the state variable z can be rewritten using $T_{p}$ as:

(12) $$z=T_{p}\;p\;$$

where the new variable p is the real-valued modal response. Substituting Equation (12) into Equation (2) gives:

(13) $$\dot{p}=\Lambda_{m}\;p+T_{p}^{-1}\;B\;f$$

It is noted in Equation (13) that $\Lambda_{m}$ is the new system matrix obtained by the transformation matrix $T_{p}$. The load term $T_{p}^{-1}\;B$ can be expressed in the form as follows:

(14) $$T_{p}^{-1}\;B=\left[\begin{array}{c} B_{1}\\ B_{2} \end{array}\right]$$

Using Equation (14), Equation (13) can be broken down into the following:

(15) $$\dot{r_{1}}=r_{2}+B_{1}f$$

$$\dot{r_{2}}=\;\Omega r_{1}+\Xi \;r_{2}+B_{2}\;f$$

The variables in Equation (15) are given as

(16) $$r_{1}=\left(\begin{array}{c} \begin{array}{c} p_{1}\\ p_{2} \end{array}\\ \begin{array}{c} \vdots \\ p_{n} \end{array} \end{array}\right)\;r_{2}=\left(\begin{array}{c} \begin{array}{c} p_{1+n}\\ p_{2+n} \end{array}\\ \begin{array}{c} \vdots \\ p_{2n} \end{array} \end{array}\right)\;\Omega =\left[\begin{array}{cc} \begin{array}{cc} -\omega_{1}^{2} & \\ & -\omega_{2}^{2} \end{array} & \\ & \begin{array}{cc} \ddots & \\ & -\omega_{n}^{2} \end{array} \end{array}\right]$$

(17) $$\Xi =\left[\begin{array}{cc} \begin{array}{cc} -2\xi_{1}\omega_{1} & \\ & -2\xi_{2}\omega_{2} \end{array} & \\ & \begin{array}{cc} \ddots & \\ & -2\xi_{n}\omega_{n} \end{array} \end{array}\right]$$

The unknown transformation matrix $T_{p}$ should be determined by Equation (12). The modal response *p* can be expressed as follows using the linear transformation matrix *W*:

(18) $$p=T_{p}^{-1}\;z=W^{T}\;z,\;T_{p}\;W^{T}=I$$

As shown in Equation (11), the mode matrix $T_{p}$ in the state space domain transforms the system matrix *A* into $\Lambda_{m}$. Equation (18) indicates that in order to determine the mode transform matrix $T_{p}$ and modal response p, the state variable z should be defined. In structural monitoring, the structural response is generally measured by accelerometers. Thus, the velocity and displacement can be converted into the frequency domain using the integrator in the time domain as follows:

(19) $$d\left(s\right)={\frac{a\left(s\right)}{s}}^{2}\;v\left(s\right)=\frac{a\left(s\right)}{s}$$

where *a*(*s*) is a Laplace transform of the m×1 acceleration vector in the *s* domain and m is the number of sensors assumed equal to the number of the main modes to be identified. Using Equation (19), the state variable and its differential state variable can be constructed as follows:

(20) $$z\left(s\right)=\left(\begin{array}{c} d\left(s\right)\\ v\left(s\right) \end{array}\right)\;\dot{z}\;\left(s\right)=\left(\begin{array}{c} v\left(s\right)\\ a\left(s\right) \end{array}\right)\;$$

The modal response is obtained by a linear transformation of the state variable or the derivative given in the frequency domain:

(21) $$p_{i}\left(s\right)=W_{i}^{T}\;z\left(s\right)\;\mathrm{or}\;\;{\dot{p}}_{i}\left(s\right)=W_{i}^{T}\;\dot{z}\left(s\right)$$

where $p_{i}\left(s\right)$ is the modal response of the i-th mode and $W_{i}$ is the 2m×1 column vector which is calculated by transposing the i-th row in the inverse matrix of the mode matrix $T_{p}$ in the state space domain. In order to force the separated mode in Equation (21) to be a real vibration mode of the system, the following conditions, suggested by Hwang [26] in a previous study, should be met [25,26]: (1) Given the constant total energy of the decomposed mode, the variance value of the modal response spectrum that can be calculated by the integral of the modal response spectrum should be unity. (2) The spectrum of the separated mode has the maxim amplitude in the vicinity of the natural frequency of the corresponding mode. 

Those conditions can be presented in form of an objective function as:

(22) $$J=S_{pp}\left(\omega_{n}\right)+\lambda \left(\int_{-\infty }^{\infty }S_{pp}\left(\omega \right)\;d\omega -1\right)\;=W_{i}^{T}\;S_{zz}\left(\omega_{n}\right)W_{i}+\lambda \left(\int_{-\infty }^{\infty }W_{i}^{T}\;S_{zz}\left(\omega \right)\;W_{i}\;d\omega -1\right)$$

where $\omega_{n}$ is the natural frequency of the target mode and λ is the Lagrange multiplier constraining the variance value of the mode response spectrum to unity. The linear transformation vector $W_{i}$ can be determined as the maximum value found of the objective function J, i.e., the derivative of Equation (22) with respect to $W_{i}$ is equal to zero:

(23) $$\frac{\partial J}{\partial W_{i}}=S_{zz}\left(\omega_{n}\right)W_{i}+\lambda \left(\int_{-\infty }^{\infty }\;S_{zz}\left(\omega \right)\;d\omega \right)W_{i}=0$$

It is noted that Equation (23) represents the eigenvalue problem with respect to the spectrum matrix of the state variable $S_{zz}\left(\omega_{n}\right),-\int_{-\infty }^{\infty }S_{zz}\left(\omega \right)d\omega$, such that $W_{i}$ and λ are the eigenvalue and corresponding eigenvector of the eigenproblem, respectively. Since the size of the response spectrum matrix is 2m×2m, the number of the linear transform vectors obtained by solving Equation (23) is also 2m. By choosing the vectors corresponding to the first two largest maximum eigenvalues, two linear transform vectors $\left[\;W_{i},\;\tilde{\;W_{i}\;}\right]$ can be determined. Each of the two neighboring modes due to the tower and the TMD produces two corresponding demixing vectors. In total, four demixing vectors can be selected and expressed in a matrix form as:

(24) $$W=[W_{1},\;W_{2},\tilde{\;W_{1}},\;\tilde{\;W_{2}}]$$

where $W_{i}$ and $\tilde{W_{i}}$ are the eigenvectors corresponding to the second largest and the largest eigenvalue of the i-th mode in Equation (23), respectively. However, it is noted that the main challenge of this approach is that even though the decomposed mode satisfies the condition of the objective function in Equation (22), the decomposed mode might not be monochromatic and can be distorted by the influence of the close neighboring mode. The main reason for this is that the neighboring mode may distort the response spectrum due to maximizing the target mode at the corresponding natural frequency while the interference between the close modes is removed if the neighboring modes are not close to each other. 

To address the mode interference issue, the objective function in Equation (22) can be modified taking the averaging spectrum into account as:

(25) $$J=\frac{S_{pp}\left(\omega_{n}\right)}{\int_{\omega_{k}-\Delta \omega }^{\omega_{k}+\Delta \omega }{\left(log\left(\frac{S_{pp}\left(\omega \right)}{S_{H}\left(\omega \right)}\right)\right)}^{2}d\omega }+\lambda \left(\int_{0}^{\infty }\;S_{pp}\left(\omega \right)\;d\omega -1\right)$$

The difference in the objective function of Equation (25) from that of Equation (22) is the added denominator term that minimizes the effect of the neighboring modes by introducing the logarithmic ratio of the power spectrum of the target mode $S_{pp}\left(\omega \right)$ to the monochromatic power spectrum $S_{H}\left(\omega \right)$ around the natural frequency $\omega_{k}$ of the neighboring mode. The monochromatic spectrum hereinafter referred to as MS is given as:

(26) $$S_{H}\left(\omega \right)=S_{O}{\left|H\left(s\right)\right|}^{2}\;,\;H\left(s\right)=\frac{s}{s^{2}+2\xi_{1}\omega_{1}s+\omega_{i}^{2}}\;$$

where the lower case s is the Laplace variable and $H\left(s\right)$ is the transfer function of the velocity response from a single-degree-of-freedom (SDOF) system which describes the target monochromatic mode. Although the damping ratio $\xi_{i}$ is unknown in this step, it can be approximated using the power spectrum of the decomposed mode evaluated by the conventional mode decomposition method. If the modal response represents the acceleration, the numerator in $H\left(s\right)$ is $s^{2}$. $S_{o}$ in Equation (26) is calculated using the natural frequency of the target mode $\omega_{n}$ as follows:

(27) $$S_{O}=\frac{\int_{\omega_{n}-\Delta \omega }^{\omega_{n}+\Delta \omega }S_{pp}\left(\omega_{n}\right)d\omega }{\int_{\omega_{n}-\Delta \omega }^{\omega_{n}+\Delta \omega }{\left|H\left(s\right)\right|}^{2}d\omega }$$

Once the variables in Equation (24) are determined, the linear transform matrix W is established in the same way as described in Equation (23). However, since the objective function in Equation (25) is nonlinear, it is difficult to derive the transform matrix W in a closed-form expression as expressed in Equation (23). Instead, the transform matrix W can be determined through optimization using the sensitivity function of the objective function to W. 

Lastly, the mode decomposition can be performed using the two objective functions presented above: Equations (22) and (25). In this study, the former and the latter are referred to as state space-based mode decomposition (SSBMD) method and the optimized state space-based mode decomposition (OSSBMD) method, respectively. In order to validate the efficacy of the proposed methods, this study examined the vibration signal from the TMD system which is one of the typical non-classically damped structures. A TMD is a passive damping device designed to reduce the dynamic response related to a particular vibration mode of the structure. As the TMD is tuned to the target natural frequency of the structure, two new modes are produced. Since those two modes are very close to each other, the system with a TMD exhibits typical non-classical damping. This study attempts to decompose the two closely spaced modes from the measured vibration signal data using the proposed techniques.

## 3. Experimental Validation of the New Mode Decomposition Method

### 3.1. Benchmark Structure and Data Acquisition

**Figure 1:**
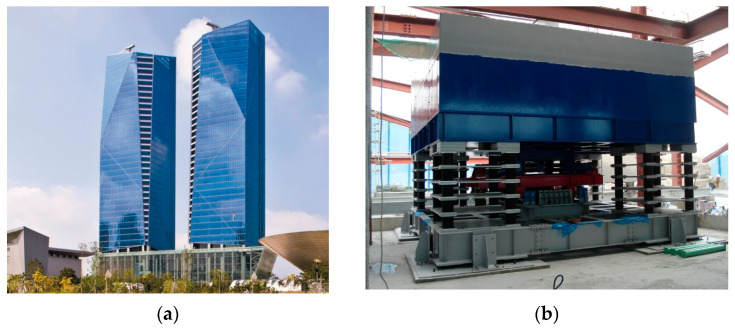
Benchmark structure: (**a**) Overview of the Posco E&C Tower in Songdo, South Korea; (**b**) 160 ton tuned mass damper.

To validate the proposed SSBMD and OSSBMD methods, a case study on a high-rise building was conducted. Acceleration responses were measured from a 184.6 m, 50,354 ton steel benchmark building, consisting of two towers located on the northwest seashore of South Korea, as shown in Figure 1a. A tuned mass damper (TMD) was installed on top of the south tower to reduce lateral vibration caused by strong wind loads, which could be amplified by the neighboring towers. This study aimed to compare the performance of two-mode decomposition methods.

**Figure 2:**
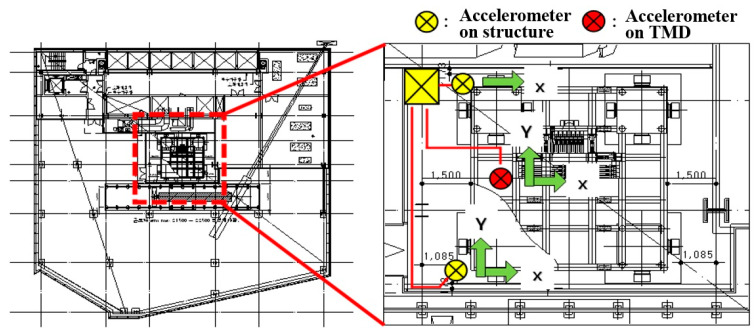
Layout of the accelerometers and the axes of the recorded accelerations.

**Figure 3:**
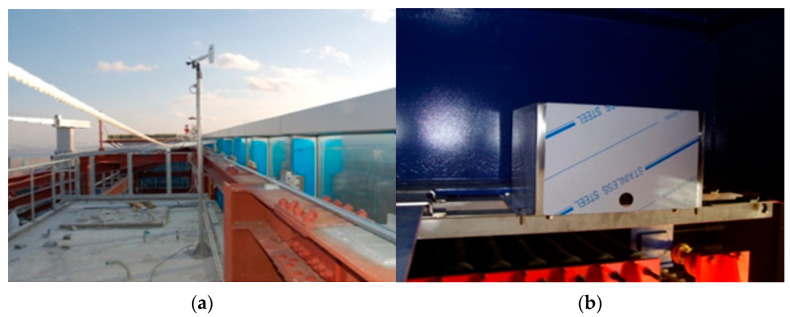
Data acquisition: (**a**) Anemometer on the top level of the tower; (**b**) the accelerometer installed on the TMD.

The response induced by wind loads on the tower and the tuned mass damper (TMD) were recorded using a monitoring system installed on the roof level of the tower where the TMD was mounted. Accelerometers were arranged in a specific layout to capture the response in the X- and Y-directions and torsion, the wind speed and direction, and the dynamic response of the TMD resulting from the tower’s motion, as shown in Figure 2 and Figure 3. The sampling frequency was 100 Hz during the 60 min measurement time, with a frequency resolution of 1/3600 Hz for the analysis.

The proposed method in this study was used to decompose the neighboring modes induced by the 160 ton TMD installed on the top of the building. The Y-direction accelerations of the structure and the TMD were selected for mode separation. Since the amplitude of the X-direction acceleration measured during the test was much smaller than that in the Y-direction, only the motion in the Y-direction was considered to identify the dynamic properties of the tower and the TMD. The results of the experiment are presented in Table 1.

Based on the identified modal properties of the structure and TMD, the complex modal matrix (Equation (4)) and real-number modal matrix (Equation (11)) were calculated, along with the demixing matrix (Equation (18)). The real modal matrix and demixing matrix were normalized to the magnitude of each vector and used as the analytical solution for comparison with the demixing matrix W calculated by the proposed method. 

**Figure 4:**
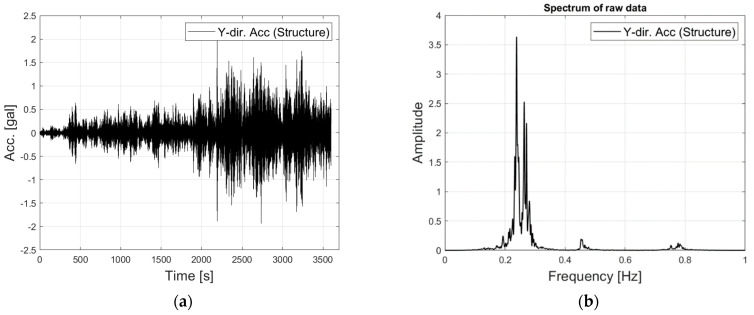
The recorded response of the tower: (**a**) Time history; (**b**) power spectrum.

**Figure 5:**
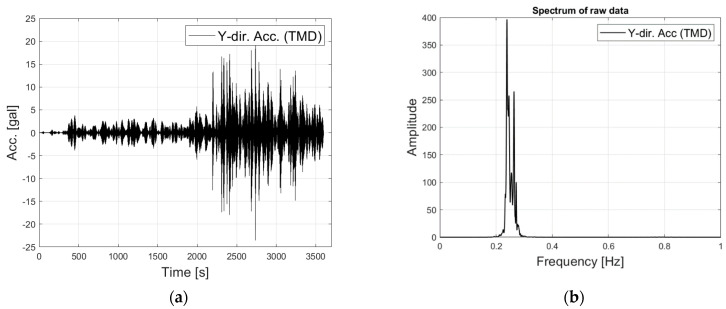
The recorded response of the TMD: (**a**) Time history; (**b**) power spectrum.

Figure 4 and Figure 5 illustrate the sampled Y-direction responses of the tower and TMD for 60 min in the time and frequency domain. As shown in Figure 4, the tower’s acceleration increased with time up to 2 gals. The power spectrum of the tower’s response identified natural frequencies in the range between 0.2 Hz and 0.3 Hz, along with peaks at around 0.5 Hz and 0.8 Hz. Two closely spaced natural frequencies were observed between 0.2 Hz and 0.3 Hz due to the tower’s natural frequency being harmonized with that of the TMD.

In contrast, the maximum acceleration of the TMD was 25 gal, which is 12 times greater than that of the tower. The TMD was designed to interact with the fundamental mode of the tower, so little influence of the higher mode was observed in the power spectrum, as seen in Figure 5b.

### 3.2. Validation of the Proposed Mode Decomposition Method

**Figure 6:**
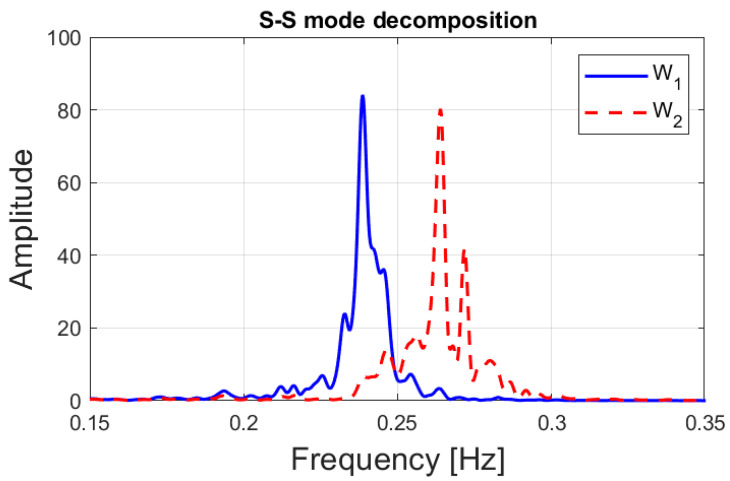
Separated modes of the structure using SSBMD.

The proposed mode decomposition method was verified using monitored response data from the tower and TMD system, and the results are presented in this section. The SSBMD method was used to decompose the closely spaced modes, and the separated modes are displayed in Figure 6. The power spectra of the modal responses corresponding to the two modes in the demixing matrix are shown in Figure 6, and it was observed that the neighboring peaks were successfully separated.

The state variables and their first derivatives need to be determined as given in Equation (20) to be used for the proposed SSBMD and OSSBMD. Hence, the measured acceleration was converted into the displacement and velocity in the frequency domain using Equation (19). As mentioned above, the state variables and differential state variables can be evaluated using the time integrator. 

Though four modes appear in the state space domain, only the power spectrum of the modal responses corresponding to the $\tilde{\;W_{1}}\;\mathrm{and}\;\tilde{\;W_{2}}$ in the demixing matrix, W, are displayed in Figure 6. 

**Figure 7:**
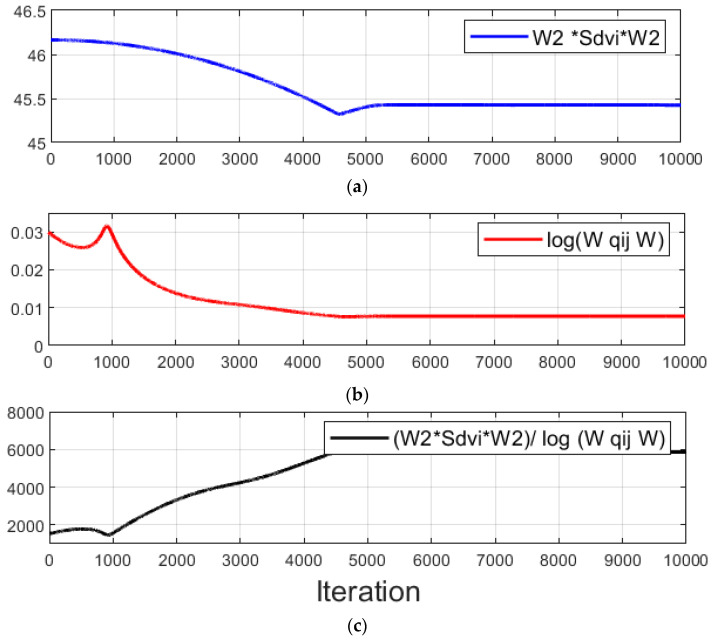
Convergence of the performance index of the OSSBMD objective function (Equation (25)): (**a**) Numerator of the objective function; (**b**) denominator of the objective function; (**c**) performance index.

Then, the OSSBMD method was used to decompose four modes, and the optimization process in the decomposition of the second mode is illustrated in Figure 7 in terms of the converging performance index. This step is to determine the demixing vector $\tilde{\;W_{2}}$. The numerator and denominator of the objective function presented in Equation (25) and the resultant value of the objective function against the number of iterations are shown in Figure 7a–c. The initial value used for the optimization through the OSSBMD was the same as that obtained by the SSBMD method. In Figure 7a, convergence is observed with a decrease in the numerator value from the objective function of the OSSBMD with the number of iterations. Likewise, the denominator value of the objective function in Figure 7b decreases rapidly and converges over the number of iterations. The introduced term in the denominator in the first term on the right-hand side of Equation (25) helped to minimize the difference between the target mode spectrum and the averaging spectrum, resulting in a convergence of the objective function of the OSSBMD method, as shown in Figure 7c. 

**Figure 8:**
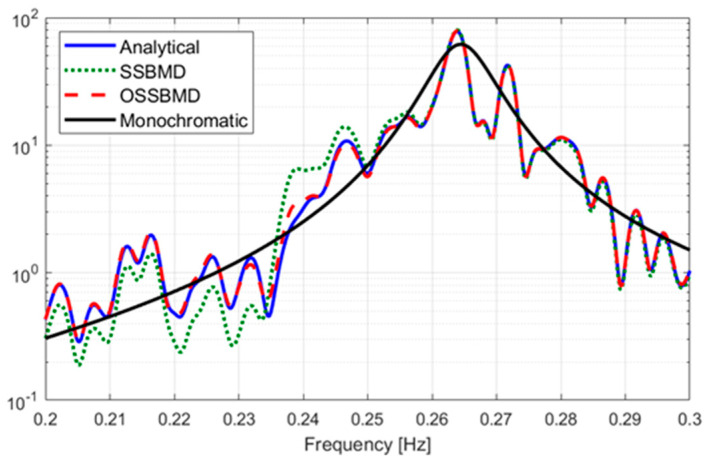
Comparison of the separated modes on a logarithmic scale.

To compare the mode decomposition performances of the SSBMD and OSSBMD methods, four modes were obtained using the optimization process. The modes corresponding to the demixing vector, $\tilde{\;W_{2}}$, are shown in Figure 8, along with the modes obtained by the analytical method from Table 1 and the averaged power spectrum of the monochromatic mode. The results showed that the OSSBMD method provided better mode separation compared to the SSBMD method and the modes obtained from the analytical method.

Figure 8 presents an interesting finding that the difference in spectrum amplitude of the separated mode by SSBMD is greater than that by OSSBMD with respect to the monochromatic mode in the frequency range of the first mode, around 0.22–0.25 Hz. This outcome is likely due to the distortion of the power spectrum near the neighboring natural frequency to maximize the spectrum amplitude at the target mode’s natural frequency. The SSBMD technique is based on the eigenproblem, which allows for transforming the spectrum in line with the target mode’s spectrum while keeping the area under the spectrum curve unchanged. It is important to note that, as depicted in Figure 8, the OSSBMD approach improved the transformation near the analytical method’s spectrum of the target mode without a significant deterioration near the neighboring mode’s frequency.

**Figure 9:**
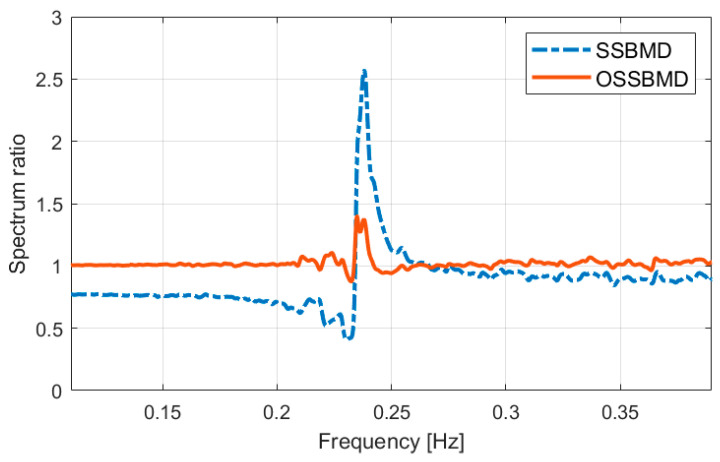
Comparison of spectrum ratio of separated mode to original mode.

This research paper shows another significant finding indicating the superiority of the OSSBMD method over SSBMD regarding the distortion and amplification of the decomposed mode spectrum. The spectrum ratio of the mode decomposed by SSBMD and OSSBMD to the analytical mode is presented in Figure 9. The SSBMD method resulted in a steep increase in ratio at the neighboring mode’s frequency and a significant decrease below the 0.22 Hz range, which can be attributed to the distortion of the power spectrum near the neighboring natural frequency. In contrast, the OSSBMD method provided a nearly identical spectrum to the analytical mode, except for a slight difference near the natural frequency of the neighboring mode. It is worth noting that although OSSBMD performs better than SSBMD, the latter is still a useful and convenient approach because the demixing matrix can be easily calculated through the eigenproblem. Moreover, the demixing matrix derived from SSBMD can be used as the initial value for the optimization process to improve the quality of the decomposed mode through the proposed method.

Table 2 presents a comparison of the demixing matrices obtained through the OSSBMD and SSBMD methods for the first and second modes, along with the normalized vectors and correlation coefficients to the analytical mode. While the demixing matrices are similar for both methods, the vectors obtained through OSSBMD have a higher correlation with the analytical solution than those obtained through SSBMD. These results support the use of the demixing matrix calculated from SSBMD for mode decomposition, followed by optimization through OSSBMD for more accurate modes.

The proposed state space mode decomposition method was validated by extracting closely spaced modes in the response of the tower and TMD in a non-classical damping system. The study found that mode decomposition using the new approach in the state space domain was more effective than using the conventional method in the MCK domain. Additionally, the OSSBMD method was shown to be useful in preventing spectrum interference near the natural frequency of neighboring modes, which is often observed in structures with damping devices.

## 4. Conclusions

The aim of this research was to assess the efficacy of an optimized mode decomposition method in the state space domain for non-classically damped structures. The proposed method was found to effectively address the closely spaced mode issue commonly encountered in structure-TMD systems by separating neighboring modes independently. However, the modal analysis of the health monitoring data from the tower and TMD revealed a limitation in the power spectrum of the separated mode, which decreased in the vicinity of the neighboring mode’s natural frequency. To overcome this distortion issue, a new objective function based on a constraint condition of the power spectrum was introduced. The result of the mode decomposition using this new objective function showed a significant reduction in interference between neighboring modes. It is recommended that a more stable algorithm be investigated for the optimization process to apply an advanced nonlinear objective function for more effective mode decomposition and structural health monitoring.

## Figures and Tables

**Table 1:** Dynamic properties of the structure-TMD system.

Type	Item	Value		
Structure	Modal mass	13,453 ton		
	Natural frequency	0.25 Hz		
	Damping ratio	0.78%		
TMD	Moving mass of TMD	160 ton		
	Natural frequency	0.252 Hz (suboptimally tuned)		
	Damping ratio	4.1%		
System Matrix A	0	0	1.0000	0
	0	0	0	1.0000
	−2.4972	0.0298	−0.0260	0.0015
	2.5070	−2.5070	0.1298	−0.1298
Complex eigenvalue	1st mode		2nd mode	
	−0.0313 + 1.4963i	−0.0313 − 1.4963i	−0.0467 + 1.6612i	−0.0467 − 1.6612i
Complex eigenmatrix	1st mode		2nd mode	
	0.0163 − 0.0602i	0.0163 + 0.0602i	0.0183 + 0.0506i	0.0183 − 0.0506i
	−0.0115 − 0.5519i	−0.0115 + 0.5519i	−0.0144 − 0.5126i	−0.0144 + 0.5126i
	0.0896 + 0.0263i	0.0896 − 0.0263i	−0.0850 + 0.0280i	−0.0850 − 0.0280i
	0.8262 + 0.0000i	0.8262 + 0.0000i	0.8522 + 0.0000i	0.8522 + 0.0000i
Real eigenmatrix (normalized)	0.0181	0.023	−0.1083	0.0982
	−0.0277	−0.0336	−0.993	−0.9937
	0.1084	−0.0982	0.0473	0.0542
	0.9936	0.9943	0	0
Demixing matrix (normalized)	−0.0332	0.0332	−0.9947	0.9936
	0.0509	−0.0509	−0.0994	−0.1071
	0.9931	−0.9925	−0.0206	0.0264
	0.1006	0.1062	0.0176	−0.024

**Table 2:** Demixing matrix of the decomposed modes.

Mode Decomposition	Demixing Matrix				Correlation Coefficient			
	$W_{1}$	$W_{2}$	$\tilde{\;W_{1}}$	$\tilde{\;W_{2}}$				
SSBMD $(W_{ss}$)	0.0546	0.2509	−0.9879	0.9889	$corr\left(W_{p,\;}W_{ss}\right)$			
	0.1415	−0.0878	−0.1138	−0.1252	0.9968	0.9792	0.9954	0.9984
	0.9825	−0.9570	0.0874	0.0718				
	0.1078	0.1160	0.0589	−0.0351				
OSSBMD $(W_{opt}$)	0.0533	0.1160	−0.9914	0.9915	$corr\left(W_{p,\;}W_{opt}\right)$			
	0.1325	−0.0495	−0.0769	−0.1030	0.9959	0.9975	0.9983	0.9989
	0.9854	−0.9856	0.0786	0.0705				
	0.0928	0.1124	0.0706	−0.0357				

## Data Availability

Data available on request from the authors.
